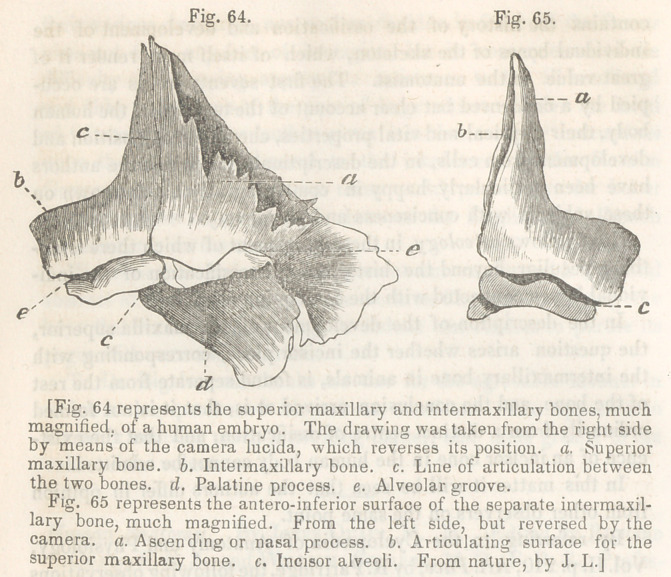# Bibliographical Notices

**Published:** 1849-07

**Authors:** 


### BIBLIOGRAPHICAL NOTICES.

*Treatise on Epidemic Cholera ; being lectures delivered under the authority of the Faculty of Medicine of Paris.* By AMBROSE TARDIEU, M. D., Adjunct Professor in the Faculty of Medicine; Physician of the Central Bureau of the Hospitals of Paris. Translated from the French, by SAMUEL LEE BIGELOW, M. D. With an appendix, by a Fellow of the Massachusetts Medical Society. Boston: Ticknor, Reed & Fields, 1849.

At the present time, when cholera is raging with more or less violence in different parts of our country, the publication of the above work seems peculiarly appropriate. Written by a physi- of high authority in France, its contents will be read with much attention by American physicians, and though its pages may not contain the disgusting boasting of the charlatan and quack, as to the discovery of some "certain cure" for Asiatic cholera, their perusal will convince us that its author has endeavored with true philosophic candor, to detail the facts connected with the causes, symptoms, pathology and treatment of this terrible disease.

For the purpose of presenting our readers with some account of the true Asiatic cholera, we shall proceed to examine in detail the contents of this book, and in doing so, we will draw from other sources, whatever may seem calculated to elucidate the pathology, treatment, &c., of this disease which threatens to invade every part of the North American continent.

In chapter 1st, our author gives us an interesting historical sketch of epidemic cholera, from which it appears that the disease is not a new one, as has been supposed by some authors, since notices of its occurrence may be found among the writings of Hippocrates, Aretœus, Celsus, Galen, &c. During the 17th and 18th centuries, we have numerous accounts of cholera epidemics, the most remarkable of which are those described by Willis, Syndenham, Bontius, Monro and others. It is only within the last few years, however, that this disease has shown a disposition to extend itself from one country to another, so that at the present day all parts of the world have been scourged with it to a greater or less extent. Since the occurrence of the three epidemics of the present century, many valuable works have been published on the subject of cholera. In addition to those cited by Dr. Tardieu, we would refer our readers to a very valuable paper, prepared in 1832 by Drs. Bell and Condie of this city. The substance of this paper may be found in Bell & Stokes' Practice of Physic. Many other articles of considerable value have appeared from time to time in the English and American journals for the years 1848–9.

Chapter 2d contains a description of the symptoms of cholera. The disease is divided by our author into two periods, the one of *collapse*, the other of *reaction.* The stage of collapse, is preceded generally by certain precursory symptoms, such as a state of indescribable suffering, rapid prostration, deep colicky pains, anorexia, diarrhœa, profuse sweats, disturbance of the senses, retardation of the pulse, &c. This condition, often accompanied by great mental depression, may last for a longer or a shorter time, when the first stage of the disease becomes established. In some cases the attack comes on without any premonition whatever.

*First period.* The precursory signs increase in severity, the vomiting and purging become constant, the discharge from the intestines may be at first serous and slightly bilious, soon, however, becoming liquid and whitish, resembling unclarified whey, or a decoction of rice, and emitting an insipid spermatic odor. In some cases the discharges are mingled with blood or bile, and even worms. The substance vomited is of a similar character. These discharges are rarely absent, and sometimes, though not always, they continue throughout the whole course of the disease. A deep pain in the epigastrium, with constant thirst and hiccough, harass the patient. Painful cramps soon come on, affecting not only the calves of the legs, but every muscle of the body. A spasmodic contraction of the muscles of the fingers and toes, is also frequently observed. The pulse is feeble, almost imperceptible; the countenance becomes hippocratic, the coldness of the surface increases very rapidly, the skin becomes blue and wrinkled, the nails livid, the genital organs retracted. The size of the body diminishes perceptibly, the eye is sunken in the orbit, and is surrounded by a bluish circle; the conjunctiva becomes pale, the cornea dull and dry, the respiration "slow and feeble, or rare and anxious;" the breath and tongue are cold, the pulse is reduced to the merest quiver. There is an arrest in the secretions, especially of urine; the voice is reduced to a whisper, the nose is cold, the surface is covered with livid blotches, the face and extremities are moistened by a viscid, cold sweat; up to a certain period, the intellect remains unclouded, but towards the close of the stage of collapse, it becomes obscure; the pulse ceases, the respiration is laborious, and death soon takes place.

The first stage of the disease is designated under the terms, cold, livid, collapsed, or asphyxiated period. It may terminate fatally in consequence of the discharges from the stomach and bowels, even before cyanosis or cramps come on: in other cases it is prolonged, when the patient will present the whole symptoms which have been described.

If death does not take place, at the close of this stage of the disease, one of two things will occur, *either* the health will be restored by a natural process, without the intervention of any new order of symptoms, or the symptoms of collapse will be replaced by those of the second period of reaction.

*Second period.* The coldness ceases to increase; the warmth of the surface gradually returns; the pulse beats fuller and stronger, becoming febrile in its character; the color of the face, the animation of the countenance, &c., become marked. Where a favorable termination is about to take place, the vomiting ceases, and though the diarrhœa may persist, the character of the stools changes; they are no longer *choleric;* the secretion of urine is re-established; the nausea, the pain, the thirst, all pass off; the pulse becomes regular, and convalescence is established. In some cases the phenomena of reaction are but slowly developed, and the patient remains for a longer or a shorter time in a most precarious and uncertain condition, which may finally terminate either in death or recovery.

When the reaction is very energetic, a new order of symptoms arise, equally dangerous to the well being of the patient, in as much as they give rise variously to apoplexy, spasms, convulsions, local congestions, inflammations, &c. &c. Or the patient may fall into a state of stupor, presenting some of the symptoms of the last period of typhus fever.

Our author states, that " swelling of the parotid glands, various eruptions of the skin, as roseola, urticaria, erythema, erysipelas, miliary vesicles, etc., manifest themselves towards the close of the disease."

The average duration of cholera is from one to three days. Its course is sometimes much more rapid, producing death in less than six hours, even without the occurrence of diarrhœa or vomiting. At other times the disease is much prolonged. The convalescence being usually slow, is accompanied by many signs of ill health, which require the careful attention of the medical practitioner.

Our author, after insisting upon the necessity of treating the early premonitory symptoms of cholera, gives us an interesting account of its individual symptoms. Diarrhœa is almost always present; sometimes continuing throughout the whole course of the disease, at others, becoming suppressed during the cold stage, notwithstanding the continuance of the other symptoms to a fatal termination. Vomiting is an early symptom; "generally it ceases before the termination of the disease, sometimes early in its course. It rarely happens that vomiting persists to the second stage of the disease." Nausea, epigastric pain, thirst, &c., are common attendants upon an attack of cholera. The secretion of urine is generally suppressed; still, in some cases, the urine remains normal throughout the whole course of the disease." In others, after having been suppressed, a renewal of the secretion has occurred, and the emission is involuntary. During the stage of reaction, the urine is freely secreted, especially where the reaction is free and regular.

One of the most distressing symptoms of cholera, consists in cramps of the different muscles of the body. They are rarely absent, and in some cases they have been the only symptom of an attack of cholera. They sometimes continue, even after life has to all appearances become extinct. Some curious cases of this kind have been cited by our author. The intellectual faculties remain unimpaired during almost the whole period of collapse; but when reaction comes on, the patient becomes stupid, more rarely delirious. Headache is not often present except during the period of reaction; the senses of sight and touch are much blunted during the collapse of cholera. There is great loss of strength; the voice is impaired; the respiration difficult; and the pulse feeble and almost imperceptible during the period of collapse. As reaction comes on, the sight, general sensation, increased muscular strength, the state of the voice, of the pulse and of the respiration undergo a marked improvement. "The perversion of hematosis, and of the course of the blood coincides with a reduction of the general temperature of the body." The skin loses its elasticity, becomes faded, wrinkled and cold. The breath is cold, being reduced 10° or 12° degrees below its normal temperature. There is a good deal of difference of opinion, in regard to the amount of reduction which the temperature of the body undergoes. M. Czermack has observed the temperature of the feet fall to 63° (Fahr.,) and he adds that below the general temperature of 74° (Fahr.) death was certain. M. Monneret, on the other, hand states, "that in nearly all the subjects, who died during the epidemic at Constantinople, in the midst of the choleric coldness or some time after it had ceased, the temperature did not deviate sensibly from the natural state." In commenting upon these discrepancies, M. Tardieu says, "however this may be, the most positive observations establish the fact, that the blood circulates but imperfectly, and that it shuns the periphery; that the respiration is imperfectly performed, and that from these circumstances, there results a considerable reduction in the temperature of the body; of which, however the patients are unconscious." As reaction returns, the warmth of the surface is recovered; the skin loses its shrivelled appearance and its cadaveric hue. Coincident with this improvement in the heat of surface, &c., the countenance becomes brighter, more expressive and more natural.

Secondary affections are frequently met with in cholera patients. In warm latitudes, the disease sometimes terminates towards the close of the stage of reaction, in a fever very analogous to bilious fevers of those regions. The secondary affections as observed in Europe, have been arranged by M. Littré in four principal classes. 1st. Where gastro-intestinal inflammations occur immediately after the period of reaction or during convalescence. 2d. When under the influence of reaction, inflammatory congestions of the organs of respiration make their appearance. 3d. Where, instead of convalescence, we have the developement of a fever resembling the typhoid fevers of the country. In other cases a genuine form of Intermittent may show itself. 4th. " But the most characteristic and formidable secondary affections are those which attack the nervous system. The cerebral congestions, so frequent during the period of reaction, are, in some cases, followed by a perfectly characterized meningitis accompanied by trismus. M. Rayer has described under the term, "*étdt cérébral cholérique*," a group of peculiar phenomena, very distinct from those of imflammation of the meninges and brain, which supervene upon the cold period. This is a sort of prolongation of that period, with a diminution or cessation of the vomiting, alvine evacuations and cramps, and the developement of cerebral symptoms—the skin continues cold or cool—the nose is cold—the tongue is yellowish and sometimes cold; if there be injection of the eyes, it is only upon their inferior parts, the pulse is feeble, the head heavy, the countenance stupid, and in some cases the tint peculiar to cholera remains. Our learned teacher also observed, in a patient who had passed the cold stage, a sort of non-febrile delirium, which continued for two or three days; and in a convalescent, a contraction of the flexor muscles of the fore arm, occurring suddenly, entirely analogous to the idiopathic spasmodic contraction of the extremities, which may be grouped with those spasms limited to certain muscular fibres, which have been described by M. Magendie under the term "*reáction febrillaire.*"

Cholera may terminate fatally, either during the first or second stage. On the other hand, recoveries take place in various ways. Sometimes the symptoms of the cold stage, rapidly disappear, as it were, by true resolution—the favorable change in some cases being accompanied by critical discharges. As a general rule, however, cholera disappears in a more gradual manner; the patient undergoing various perilous struggles before convalescence is established. The convalescence, occasionally rapid, at times progresses very slowly, the individual suffering for a longer or shorter time from continued debility, both of mind and body—from irregular appetite—from gastralgia, &c., &c. It has been observed, also, that the constitution of the individual sometimes undergoes an entire change, in consequence of an attack of Asiatic cholera.

In regard to a second attack, our author makes the following remark: "Without asserting that a grave attack of cholera predisposes to a second, it should be borne in mind that one is not completely exempt from another attack. The most recent observations, however, show that this is a very rare occurrence."

Our author makes several varieties of cholera. 1st. *Grave*

*Cholera*, the symptoms and progress of which have just been described. 2d. *Cholerine,* a mild form of the disease, marked by the precursory symptoms of the grave form, and though cramps, vomiting, and purging may be present, the disease never passes into the stage of asphyxia or collapse. 3d. *Foudroyant Cholera*, occasionally met with, when the individual is suddenly attacked, with or without premonition, with all the symptoms of collapse, from the effects of which he dies in one or two hours.

4th. *Paralytic Cholera* described as follows by Magendie: " The access is in general very slow; the patients feel nothing but an excessive weakness and loss of appetite. In the course of eight days, they fall into a state of extreme moral and physical depression, the muscles of the face become paralyzed, those of the limbs are in a state of complete resolution, the intellect loses all activity, and death occurs in the midst of this general annihilation of the powers." In this form the dejections are often wanting; the abdomen is distended by the liquids contained in the intestinal canal, the muscles of the abdomen and intestines being incapable of ejecting them.

In chapter 3d we have an account of the post mortem appearances. The exterior of the body of an individual dying of cholera, varies but little from the appearance it presented during the stage of collapse—there is the same emaciated, wrinkled appearance of the surface—the skin is livid—the muscles sometimes rigid even before the warmth of the body has disappeared. The body cools very slowly, and at times the face and hands become warmer eight or ten hours after death than they were at the time of its occurrence. The condition of the intestines is very peculiar. The peritoneal surface is, "*in all cases*," sticky and shining, covered with a substance of extreme viscidity. The vessels of the mesentery are gorged with blood—the calibre of the intestinal canal is more frequently increased than diminished. In the interior of the intestines, we find a greater or less quantity of the fluid which passed from the intestines during life. In the stomach, this liquid is either fluid, like water, or thick, in consequence of its being mingled with a very viscid substance. In the upper part of the intestine the fluid is of a milky consistence, frequently gray, yellow, greenish yellow or white—sometimes reddish—but rarely livid. In the lower portion of the intestine it is livid or bluish or chocolate color and of remarkable fluidity. In a word, as we descend in the intestines, the fluid increases in color but diminishes in consistence.

Upon a close examination, we may discover floating in the intestinal fluid, a denser substance—easily coagulated and at times adhering to the mucous surface of the intestinal canal. This substance, according to M. Contour, is soft and grayish—of the consistence of paste—and capable of being easily removed from the mucous membrane of the intestine. Contour and Monneret consider that this substance constitutes the coagulated portion of the choleric fluid.

The mucous membrane of the intestines, after the above described coagulable substance has been removed, is variously changed as to color. Most usually it presents a more or less bright red color, disposed in bands or points—or it may be livid or black. This discoloration may be made to disappear by injecting water into the gastro-epiploic arteries, for the purpose of washing out the blood, which has remained stagnant in the vessels of the mucous membrane. This fact would seem to show, that the change of color was due to congestion and not inflammation of the tissues. As to thickness and consistence the mucous membrane remains perfectly normal—nor is it found " inflamed, softened, ulcerated or gangrenous, according to M. Bouillaud and M. Bonnet, except in cases where, the disease having been prolonged, a secondary inflammation has supervened." The villi of the intestines are hypertrophied and prominent—and the solitary and agglomerate glands are constantly found remarkably developed. Throughout the internal surface of the œsophagus, stomach and intestines, are found " little hard, opaque, oval, ordinarily dull white bodies, the size of which varies from that of the point of a pin to that of a very small pea, and sometimes so numerous and near together that the whole mucous membrane seems to be covered. They rest often upon a more or less injected base, and when incised flatten down, leaving only a small elevation." This vesicular eruption termed by MM. Serres and Nonat *psorentérie*, is absent in a certain number of cases of epidemic cholera and cannot therefore be regarded as characteristic of the disease.

In a paper published by Dr. Horner in the American Journal of Medical Sciences, the following alterations of the intestines have been noticed in Asiatic cholera. "*First*, A copious vesicular eruption, entirely distinct from the tumefaction of villi, muciparous follicles, or glands, and which pervades the whole canal. *Second*, A lining membrane of coagulable lymph, which exists in the small intestines at least, if not in the stomach and colon also, and resembles in texture and mode of adhesion, the membrane of croup. *Third*, Vascular derangements and phenomena, which are confined almost exclusively, if not entirely, to the venous system. *Fourth*, An exfoliation of the epidermic and venous lining of the alimentary canal, whereby the extremities of the venous system are denuded and left patulous."

The organs annexed to the intestinal canal, do not exhibit any particular alteration as connected with cholera.

*Organs of Respiration.*—Upon the surface of the pleura, we find the same deposition of the stringy and glutinous substance, which was found upon the peritoneal coat of the intestines. The lungs are often healthy though its parenchyma and the mucous membrane lining the bronchial tubes are frequently much congested. Inflammations of the lungs occurring during the stage of re-action are to be regarded as secondary affections—the existence of which throws no light upon the pathology of Cholera.

*State of the nervous centres and of their enveloping membranes.* The sinuses of the brain and spinal marrow are almost always found gorged with black blood—occasionally coagulated and adherent to the sides of the blood vessels. The arachnoid membrane is coated with a sticky viscous substance. In the spinal canal this membrane is found covered over with "small whitish irregular cartilaginous granulations from the diameter of a mustard seed to that of a lentil, which, examined under the microscope, appeared to have a fibro-cartilaginous structure." The pia mater is congested and infiltrated with serosity. Depositions of plastic lymph and ecchymosed spots are sometimes observed on the interior of the blood-vessels. The nervous substance is frequently healthy, but occasionally its consistence is increased. It is also much congested. The ganglionic system of nerves, presents no alterations worthy of remark.

The muscles of the body are congested, and retain sometimes the sticky feeling of all tissues when congested with choleric blood. M. Begin has noticed a reddish or brownish discoloration of the bones and teeth of those who die in the stage of collapse. The kidneys are congested, but in other respects their structure is unaltered. The bladder is contracted and contains no urine. The alterations in the blood are remarkable, and may be summed up as follows: it is very dark black and viscous; very analogous to varnish, when exposed to the influence of the air it becomes but slowly oxygenated. It coagulates, but the separation of the clot and serum is very incomplete; the globules do not seem to be materially changed in form as has been asserted by Donnê. Hermann states that they are rapidly destroyed after death. In the blood of those dying of cholera, there is a marked diminution in the proportion of the water, neutral salts, fibrin and albumen. Some observers, however, assert that the fibrin and albumen are increased in quantity. Dr. O'Shaugnessy considers it doubtful whether a large amount of urea be found in the blood even where there has existed suppression of urine. Still urea has been found, not only in the blood, but in the bile of cholera patients. The serum of the blood is not as strongly alkaline as in a state of health. In view of these changes in the blood, we are not surprised to find that the discharges from the bowels during the existence of cholera, contain a large amount of the carbonate, muriate, acetate, phosphate and sulphate of soda, coagulable lymph, mucus and water. There is no material alteration in the structure of the organs of circulation. The right side of the heart and the venous system are gorged with blood, while the arteries are found empty. The air expired from the lungs, contains but little if any carbonic acid, and M. Rayer has satisfactorily proved "that the air expired by those laboring under an attack of cholera, who do not present the external character of asphyxia, contains nearly the same proportion of oxygen as the air expired by healthy individuals, and that the air expired by those cholerics in whom the external characters of asphyxia exist contains considerably more oxygen than that expired by persons in health." All these facts exhibit forcibly a very great disorder in the process of sanguification and circulation in those laboring under Asiatic Cholera.

In some microscopic investigations made by Dr. Ludvic Boehm, of Berlin, the following points have been noticed. 1st. That the main pathological alteration of the mucous membrane, consists in a loss of its epithelium by desquamation. This process is accurately described, and is supposed to commence in the inner portion of the ileum. The points where the desquamation has commenced, present a whitish color and a velvet-like appearance, as if the villi were unusually raised above the surface. One of these villi when examined microscopically is found to have its epithelium thickened and divided by concentric lines showing the beginning of the process, by which the epithelium is reduced to its elementary condition of cylindrical cells, which are soon entirely detached and thrown off. The mucous surface from which the epithelium has been detached is covered with small white depressions, which mark the original attachment of the epithelial cells. Desquamation in some cases takes place by the formation of bullæ, composed of the epithelium detached from the villus, and thrown off from it in the form of little sacs, shaped like the villi from which they have been removed. In one or the other of these modes the desquamation is accomplished. Sometimes large flakes of epithelium are found, bearing *on one side* the impress of the prominent villi—*on the other* the villous foramina may be seen. The mucous membrane is left abraded—the villi become reduced in circumference—more flaccid and subjected to a complete process of maceration. The mucous crypts are destroyed, the mucous membrane exhibits a number of figures, from which an extravasation of blood takes place.

This process of desquamation occurs so rapidly that Dr. Boehm asserts that he has seen it accomplished in the bodies of patients, who six hours before death had been in health.

Dr. Boehm also examined the contents of the stomach and bowels microscopically. This fluid when poured into a convenient vessel soon separates into two portions, *the one* semi-transparent constitutes, according to this observer, the true pathological secretion. *The other* is thick and composed of epithelial cells. The more violent the disease has been, the clearer is the upper fluid, the whiter the sediment, and the more perfect the cells. The contrary is true where the disease has been lingering. The contents of the stomach and bowels vary with the amount of epithelium and the condition of its cells—so that it may be found either creamy, purulent, milky, gruel-like or mucous.

3d. The secretions from the kidneys, liver, salivary glands, tissues of the eye, &c., are much diminished in cholera. In the biliary ducts, the epithelial cells are thrown off, the contents of the stomach and bowels consist, not of bile, but of these cells, tinged with the coloring matter of bile. In one third of the cases, no bile is found in the stomach or bowels; in another third the bile was generally effused when the patient died; in protracted cases there was a superabundance of bile thrown out.

The sediment of the matter vomited, is dark, nearly black, and when treated with nitric acid, becomes of a reddish-brown color, changing to green, when dissolved in caustic potash. This sediment is regarded by Dr. Boehm as an altered condition of bilious pigment.

4th. Intestinal hemorrhage is rare, but fatal. The blood contains few corpuscles, and these much altered in form. The fæces have also been examined by Dr. Boehm, and are found sometimes tinged with bile, but more generally consisting of the rice water description.

5th. The urine is found to contain the epithelial cells of the mucous membrane of the uropoietic organs.

6th. An oily fluid is found in the intestinal villi of cholera patients. Besides this a vegetation similar to *saccharomycis cerevisiæ*, is found among the contents of the intestines of cholera patients.

7th. The mucous crypts, whose apertures in the healthy subject are seen as black points, are, in cholera patients, found filled with white corpuscles. When these are removed, the orifices are found perfectly healthy, though somewhat enlarged. The desquamation of the epithelium of these crypts takes place in a very peculiar way, thus—when one layer is thrown off, it is replaced by a new one, which in its turn is extruded. The mucous gland left behind, after the desquamation, resembles very much a villus, in consequence of the cylinder of epithelium which protrudes from the orifice of the former. For this reason some authors have stated that the number of villi was much increased.

8th. The capsule of the glands of Peyer, when examined microscopically, are found softened and ulcerated at their apices, and through these ulcerated points the contents of the glands escape. The glands lose their granular shape, becoming more elevated in consequence of an exudation which takes place in their subjacent cellular tissue. There is no real ulceration of these glands. In young children they present a wrinkled appearance. The solitary glands are more distinct than usual, in consequence of the deposition which takes place in their subjacent cellular tissue.

These observations of Dr. Boehm, confirm, to a very great extent, the conclusions of Dr. Horner, which we have extracted above.

Chapter IV. contains an interesting account of the progress of the various cholera epidemics, to which we would refer our readers.

In chapter V. the causes of cholera are discussed. Everything connected with this subject is exceedingly obscure, still the various theories in regard to the causes which may produce cholera, have been arranged by our author as follows: 1st. Telluric influences, such as peculiar geological constitutions, earthquakes, &c., have been thought to occasion epidemic cholera. Such opinions are, however, in direct contradiction with many well attested facts. 2d. Atmospheric and meteorological conditions and phenomena have been classed among the causes of cholera, but these theories are based rather upon ingenious hypothesis, than upon observation and experience. Indeed, the best instituted experiments have clearly proved that there is no appreciable alteration in the atmosphere, either as it regards its density, temperature, electrical condition, &c., which will satisfactorily account for the developement of cholera under all circumstances. In the work of M. Tardieu and elsewhere, our readers may find some very interesting observations upon the electric and magnetic state of the terrestrial globe, in connection with cholera epidemics. Although cholera may occur in dry cold weather, yet it is probable that moisture, conjoined with elevation of temperature, offer the most favorable conditions for the propagation of the disease. Those individuals, living in low and damp habitations, whose diet was scanty and of bad quality, whose conduct was irregular and dissipated, appear, as a general rule, to have suffered more than those living under an opposite condition of things. Nothing is more important then, than a strict attention to the hygienic condition of a community for the purpose of avoiding serious visitations of this terrible epidemic.

From the statistics which have been collected, it would seem that though no age is exempted from attacks of cholera, yet it rarely occurs in very young children. Females appear as liable to the disease as males, and those persons previously debilitated by disease, are more likely to be attacked than those in full enjoyment of good health.

Mental affections, moral emotions of a depressing nature, are said to predispose to this disease. An inordinate dread of an attack of cholera seem to favor the development of the disease. On the other hand, the fact that insane persons are as liable to this disease as others, goes to show that general mental emotions have not the effect which has usually been attributed to them.

To discuss the question of contagion would extend this abstract of the work before us beyond the limits of this journal. We shall therefore merely state, that the profession is divided upon this question. Mr. Tardieu may be classed among the non-contagionists, but we think some facts brought to light by Dr. Graves and others, present us with some strong reasons for believing in the contagiousness of the disease. It is, however, a very doubtful question, and in drawing our conclusions, we must remember that one positive fact, proving its propagation by contagion, is worth thousands of a negative character. In a word, it must be admitted that we know little else of the causes of cholera, than that there is some principle of peculiar character, which, when introduced into the system, produces the disease known as malignant cholera. As to the nature of this poison, its source, &c., we absolutely know nothing.

In chapter 6th, M. Tardieu treats of the diagnosis and prognosis of cholera. Asiatic cholera may, in the early stages be confounded with sporadic or bilious cholera, or with severe cases of poisoning, or with gastro enteritis, or with indigestion, asphyxia, and the plague. But where Asiatic cholera has reached its acme, *the period of collapse*, all difficulties of diagnosis will disappear; the more especially if the cases occur during an epidemic of the true Asiatic disease.

The prognosis of course, will depend very much upon the form of the disease; upon the gravity of the symptoms and the rapidity with which they reach the acme of their intensity. The prognosis also will depend upon the gravity of the existing epidemic, and upon the hygienic condition of the community attacked.

Chapter 7th contains an account of the treatment required in a case of cholera. Our author remarks, that "different indications for the treatment of epidemic cholera result from a knowledge of the causes and analyses of the symptoms peculiar to each of the periods of the disease. The first order of indications will relate to the prophylactic treatment; the second, will furnish us the most proper means to be employed against the prodromes, the cold period and the period of reaction, complications and secondary affections, and finally against the accidents of convalescence.

*Prophylactic treatment.*—The prevention of an attack of cholera involves considerations of the highest importance, as it regards private and public hygiene. Individuals should be advised to expose themselves as little as possible to the influences of moist and illy ventilated localities. The food should be sufficient in quantity, of good quality, and well cooked. All excesses in eating, drinking, exercise, &c., should be avoided; but unless the individual has been addicted to such excesses, no change need be made in his habits of living. The effects of cold and dampness should be carefully guarded against, and for this purpose our author advises the wearing a flannel band around the abdomen next to the skin. Among the rich, the excessive indulgence in richly seasoned food, high flavored wines, &c., should be avoided, while the poorer classes should be cautioned against eating crude, indigestible vegetables and fruits, and the too free use of intoxicating liquors. In a word, everything should be proscribed calculated to disorder the digestive apparatus, and at the same time the fears of the patient, in regard to an attack, should be as much quieted as possible.

*Treatment of the Prodromes.*—This is all important, since the neglect to correct the morbid condition of the system, during the precursory stage, is, in our opinion, one of the chief reasons of the very high mortality of epidemic cholera. The physician is not called in, till these premonitory symptoms have passed away, and the patient has become wholly or partially collapsed,—a period of the disease in which the organic functions are so materially impaired that the system fails to respond to the use of the most powerful remedies. The medicines administered are not absorbed, and consequently they can exert no action whatever upon the disease. On the contrary, appropriate treatment during the precursory period will, in an immense majority of cases, be followed by an arrest of the disease. Our author classifies these premonitory symptoms under two heads. 1st. Where the digestive organs are disordered. 2d. Where the prodromes consist "in a peculiar depression of spirits, heaviness of the head, vertigo, with other nervous symptoms various in number."

Where the digestion is difficult, accompanied by abdominal pain, constipation, furred tongue, bitter taste in the mouth, &c., it will be necessary to prescribe a dose of blue pill or calomel at night, followed by a saline purgative in the morning. This course must be continued till the symptoms above related have been removed. An emetic of ipecacuanha will sometimes be serviceable in these cases. The patient must be restricted to farinaceous articles of food. On the other hand, where the patient is affected with diarrhœa, colicky pains, &c., especially after each meal, the diet should be rigidly restricted. Opium administered alone, or conjoined with blue pill or calomel, if the condition of the evacuations call for their use, will usually arrest the diarrhœa and relieve the pains. Cataplasms over the abdomen, or leeches to the anus, will be found valuable adjuvants in the management of these cases. If the diarrhœa persist, notwithstanding the above treatment, other astringents may be employed, as rhatany, catechu, kino, sugar of lead, &c. The last mentioned remedy is probably the best. Dr. Graves considers it a most valuable medicine in the treatment of Cholera. It may be combined with opium and calomel or blue pill.

"The precursory phenomena of the second order, that is to say, vertigo, nervousness, general lassitude and muscular weakness, require a different treatment, which, to succeed, ought also to be applied on their first manifestation. The patient should immediately abandon all occupation; if robust, and particularly if there be plethora, bleeding should be practised, and may be with benefit repeated after a short interval; under opposite conditions, a tepid bath or affusions of warm water may be prescribed, followed by frictions with warm flannel, repose in bed, the use of diaphoretic drinks, such as tea, infusion of peppermint or of balm, and all the means promotive of perspiration."

Our readers will find in Bell & Stokes' Practice some admirable remarks from Dr. Bell in regard to the treatment of the precursory symptoms of cholera.

*Treatment of the first period.*—The commencement of this stage of cholera is marked by repeated evacuations, both from the stomach and bowels, which, with other symptoms already mentioned in the description of the disease, rapidly increase till the supervention of the algide or collapsed period. Now, how are these early symptoms of the period of collapse to be managed? What is the pathology of cholera? Of its essential nature are we not entirely ignorant? Some have considered it a disease of the ganglionic system of nerves, but these, when examined, are found in the majority of cases to be perfectly healthy. Others regard it as a form of gastro-enteritis. Others as a disease resembling very closely congestive fever, or the cold stage of an intermittent. Many other theories might be mentioned, but the limits of this paper will not permit us to go farther than to say, that none of them satisfactorily account for the phenomena of true epidemic cholera. It is a disease produced by some poison which, when introduced into the system, gives rise to a certain train of symptoms indicating serious alteration in the functions of digestion, circulation and respiration, and in some portions of the nervous system, as is indicated by the spasms, though the intelligence is usually clear. The effect of this poison upon the tissues of the body has already been noticed, but the physician who supposes that in these structural lesions he has found the essence of the disease, is as far wrong as he who imagines that the pustular eruption constitutes the true nature of small-pox. They are only consequences of some agent, of whose nature and mode of action we are entirely ignorant. Ignorant of this, our treatment of cholera has been, and still is, very empirical. The morbid alterations guide us but little in the treatment of this horrible disease. Still, our skill in its management has been much improved by the patient investigation and the well conducted experiments of many able physicians; and although the mortality is still very great in some cases, yet, in the language of Dr. Griffin, (who has investigated the subject of the benefits of the medical treatment of cholera,) we must admit, "that without medical treatment, every person attacked with it will fall into collapse, although they may not eventually die; and that such as recover, do not do so by an arrest or cessation of the disease, but by struggling through and outliving all its stages."

Although the pathology of cholera is but little understood, we agree with Dr. Bell, in thinking that experience has taught us

"that a sedative and evacuating course is much more entitled to our confidence than a stimulating one," during the early part of the first period of the disease, and previous to the developement of collapse. Where we have reason to suspect that the stomach is loaded with some indigestible substance, emetics will be found serviceable. Of this class of remedies, ipecacuanha is probably the best. Some practitioners prefer sulphate of zinc, or tartar emetic, or mustard or salt. Emetics not only clear the stomach of its offensive contents, but aid in securing a discharge of bile, in arresting the vomiting and purging, and in restoring warmth to the cutaneous surface.

Bloodletting is a remedy relied upon by some as the only means of effecting a cure in cholera. In the early stage of the first period, or even during the premonitory stage, it may be employed with great advantage. If there be fulness of the bloodvessels, with abdominal pain, headache, vertigo, nausea, vomiting, &c., in the early stage of cholera, bleeding, so as to produce a positive effect upon the system, will be found a most valuable remedy. But venesection should not be employed after collapse has come on; for in this case, besides the difficulty in making the blood flow from the veins, we feel sure that the loss of blood could do no possible good, and most probably would result in positive harm to the patient.

Calomel has been, and still is, a favorite remedy in the treatment of the early stage of the first period. It serves to tranquillize the system, allaying the gastric and intestinal irritation, and producing a powerfully beneficial effect in restoring the various secretions to their healthy condition. If given early in the disease, the alvine evacuations, the cutaneous secretions, &c., all undergo marked improvement. The calomel may be given alone, or combined with opium, and where the diarrhœa persists, with the acetate of lead. Although M. Tardieu says but little in regard to the benefits derived from calomel and opium, the testimony of many practical physicians is most strong in its favor. Mr. Corbyn, in India, says, "calomel in doses of from 15 to 20 grains is a sedative, and has the singularly good qualities of immediately stopping violent vomiting and purging, removing spasmodic irritability, producing tranquillity of mind, exciting the secretion of the liver, and preventing the progress of inflammation."

The same views are entertained by Craigie, Annesley, and others who practised in India, during the prevalence of the cholera. In this country, similar views are entertained by many of our most distinguished physicians. Dr. Drake, of Cincinnati, says, " but the chief reliance at last was on calomel and opium, or calomel alone. To be successful, it was necessary to administer them, especially the last, in large doses, and in powder with sugar, so as to promote their rapid diffusion over the surface of the stomach. There is not, I presume, a physician in Cincinnati, who cannot testify to the efficacy of this practice. It was worth every other therapeutical means, both internal and external. The most violent vomiting would cease whenever the stomach could be brought under the influence of this compound, or of the calomel uncombined; and a speedy return of the suspended secretions of the liver and skin generally followed, after which the patient commonly recovered."

Dr. Griffin, who had charge of St. Michael Hospital, Limerick, makes the following conclusive remarks in favor of the calomel treatment. "It is now at least evident, that by the judicious application of one remedy, we can control the disease, or arrest its progress, in 84 cases out of 100, if the patient be placed under our care before the pulse has ceased at the wrist; and if, after that, no more than two or three can be saved out of ten, it is only to be considered that the stage of collapse in cholera is like the stage of muttering delirium and floccitation in fever—the almost fatal conclusion of the disease." We might cite other authority in support of the above opinions, but do not deem it necessary. We would, however, refer our readers to Dr. Bell's remarks upon the treatment of cholera, for a most able exposition of the therapeutical effects of calomel in diseases generally, but especially in the early stages of cholera.

Opium is another valuable remedy in the treatment of cholera. Its power to check diarrhœa, to relieve pain, and to promote an equalization of the circulation, are so well understood, that it is unnecessary to dwell upon its well established virtues as an adjuvant in the treatment of cholera. Camphor may also be given for the purpose of checking diarrhœa, and promoting gentle diffusive stimulation.

If there exist much abdominal pain with painful cramps, their relief may be attained by cups, leeches, and sinapisms, either over the abdomen or along the spinal column. Cold water acidulated with nitric acid, as recommended by Annesley, or lumps of ice, may be given for the purpose of relieving the intense thirst with which the patient is harassed.

*External Medication.*—Dr. Bell after noticing the great discrepancy which at first existed, in regard to external remedies, among medical men, says, " towards the last, however, the fact was forced on the attention of medical men generally, that irritating agents were not serviceable either externally or internally, and that other means must be had recourse to for the relief of the neurosthenia of the skin, analogous to that of the digestive mucous surface." Among the sedative means of allaying neurosthenia, excitement, and cramps, are baths, ranging variously from the freezing point to within a few degrees of blood heat, so as to suit the habits, constitution, and excitement of the individual. In some cases dry heat would seem preferable, producing a more favourable influence when seduously persevered in.

*Stimulants.*—The milder kind of stimulants are the best, as carbonate of ammonia, oil of turpentine, capsicum, &c. From the power which the sulphate of quinine possesses in dispersing internal congestions, we would think it a valuable medicine, either alone or combined with the calomel and opium. Many other remedies might be mentioned, as applicable to the treatment of cholera previous to the collapse, but our remarks have been already so much extended, that we must pass to the treatment of the collapsed stage of the disease.

From the stage of collapse there is but little chance of recovery, and many remedies of a very opposite therapeutical character have been resorted to without any substantial benefit. The difficulty consists in the fact that all the functions of organic life are almost entirely suspended, in some cases destroyed. Medicine may be poured in, but not being absorbed it can effect no favourable influence whatever on the system. Powerful external and internal stimulation has been tried, generally without any good effect. On the other hand, the cold douche or frictions with ice, and venesection have all been had recourse to, but in a large majority of the cases, the patient has sunk in spite of every thing. In a case so desperate, we may successively try the various remedies which have been suggested, remembering, however, that even in this stage, stimulants of great strength are hardly admissible, the more moderate ones being preferable.

Astringents are said by some to be the most approved internal remedies during the collapse of cholera, and for this purpose the acetate of lead, so valuable in the stage of diarrhœa, the sulphates of copper and zinc, may be resorted to. Dr Mackintosh has used strychnia, and with marked benefit, in checking the diarrhœa and shortening the stage of collapse. The patient should be allowed very cold and acidulated drinks, as he may desire them. The period of reaction must be treated by the use of sedatives and antiphlogistics, employed with an energy just in proportion to the degree of reaction which presents itself.

During convalescence the greatest care should be taken to correct any deviations from the healthy condition by appropriate medication and proper restriction of diet, &c.

In chapter eighth, Mr. Tardieu notices the sanatory measures which should be adopted by the public authorities. 1st. The isolation and sequestration of cholera patients is not necessary, it appearing that the disease forces itself every where in spite of the strictest quarantines and sanatory cordons. 2d. The attention of the public authorities should be directed to the importance of cleanliness, proper ventilation, &c. &c. The disinfecting agents, chloride of lime, camphor, &c., are regarded not only as useless but hurtful, and in support of this opinion, M. Tardieu appeals to the following judicious remark of M. Monneret, who says, " how many persons there are who prefer to swallow a drug sold by an empiric, or to submit themselves to some singular or ridiculous practices, or to carry a specific, or infect with some rank odor the atmosphere they breathe, rather than regulate themselves and their hygiene in a manner conformable to reason and the laws of nature." 3d. The public should be liberal in supplying the poor with good healthy food, sufficiently warm clothing, proper shelter, &c. In regard to the question, whether temporary asylums should be provided for the sick, or whether the succor should be carried to their various dwellings, our author decides in favor of the former plan, not from any fear of contagion, but in consequence of the difficulty of conveying appropriate assistance to the ill ventilated and filthy habitations of the poor of large cities. Under the head of "Instructions," our author gives insertion to the "instructions upon the cholera, addressed to the inhabitants by the Russian Government," which contains many useful precautions for better security against cholera. Also, those of the English and French Governments on the same subject.

We have thus endeavored to present our readers with a faithful abstract of M. Tardieu's valuable work on Cholera—indulging the hope that we have said enough to induce our readers to peruse its pages with care and attention.

To Dr. Bigelow our thanks are due for the agreeable manner in which he has performed his task as translator. The Appendix to the work contains much interesting matter, and adds greatly to the value of the volume.

---

*Human Anatomy.* By JONES QUAIN, M. D. Edited by RICHARD QUAIN, F. R. S., and WILLIAM SHARPEY, M. D., F. R. S., Professors of Anatomy and Physiology in University College, London. First American from the Fifth London Edition. Edited by JOSEPH LEIDY, M. D. In two volumes, with over five hundred illustrations. Philadelphia: Lea & Blanchard, 1849.

Another valuable addition to the systematic works on anatomy, has been offered to the profession in this country, by the enterprising publishers of this American edition of a standard and well known English work. A work on special anatomy, obviously offers but little scope for critical analysis or review. It is a science of facts. General anatomy, however, seems to present more field for difference of opinion, as is evidenced by the conflicting statements so often made by micrographers. In the work under notice, this department seems to have obtained special distinction. The whole section of the original work of Dr. Sharpey having been re-written, it will be found to be amply discussed and fully brought up to the knowledge of the day. This work is moreover the first systematic treatise in the English language, that contains the history of the ossification and development of the individual bones of the skeleton, which of itself must render it of great value to the anatomist. The first seventy pages are occupied by a condensed but clear account of the textures of the human body, their physical and vital properties, chemical composition and development from cells, in the description of all which the authors have been particularly happy in combining all that is known on these subjects with conciseness and perspicuity.

Next follows *osteology*, in the arrangement of which there is nothing peculiar beyond the history of the ossification of the individual bones connected with the description of each.

In the description of the development of the maxilla superior, the question arises whether the incisor piece, corresponding with the intermaxillary bone in animals, is found separate from the rest of the bone, and the conclusion arrived at is, that it is not formed ordinarily from a distinct centre of ossification, and that the existence of an incisor bone in the human body cannot be admitted.

In this matter it will be seen that the authors differ in opinion from other observers on the same point.

By referring to the Cyclopædia of Anatomy and Physiology, Vol. II. p. 210, Art. *Face*, by R. Partridge, the following observations in relation to this bone will be found. "In the mature human fœtus, no sign of this bone exists, but in examining the skulls of fœtuses about the third or fourth month of pregnancy, we observe it perfectly distinct from the maxillary bone. It sometimes happens that at more advanced periods, whether of intra, or extra-uterine life, evidence of the separation of the intermaxillary bone exists, and as Meckel says, we often find a transverse narrow '*lacuna*' on the vault of the palate, extending from the internal incisor, to the anterior palatine foramen. According to Weber, however, who examined the extensive collection of fœtal skeletons belonging to Professor Ilg, in Prague, the intermaxillary bone was distinct only in those who had double hare-lip. He considers, however, that the intermaxillary bone readily separates when the skull of a child of one or two years old is placed for some time in dilute muriatic acid."

The American editor has arrived at the same conclusion, from observations conducted by himself, and entitled to every confidence. He says:

The intermaxillary bone as a distinct piece in man, I have detected existing in the embryo of about the ninth or tenth week, (a good deal of uncertainty existing relative to the age of embryos, I will further add that it measures 1 inch 11 lines from heel to vertex, the lower extremities being stretched out.) At this period ossification has already advanced in the superior maxillary and intermaxillary bones sufficiently to give them a determinate form, and have the appearance, when magnified, as represented in figures 64 and 65, which were taken from the specimens by means of the camera lucida. The greatest breadth of the two bones in apposition is one line and two-thirds, the greatest height, being at the ascending or nasal process, is one line. They present a facial portion, consisting of the ascending or nasal process, and part of the body of the bones; an alveolar ridge and groove, and a palatine process projecting backward from the superior maxillary bone. The two are easily separable at this period, and the articulation passes through the alveolar ridge, at a point corresponding to the separation between the incisor alveoli and the canine alveolus, and extends transversely inwards behind the incisor alveoli and vertically upwards, dividing the nasal process into two nearly equal portions. On the posterior surface of the nasal process, the articulation is at the bottom of a comparatively deep and wide groove. The preparations exhibiting these points, which have been the subject of so much discussion, I have carefully preserved, and upon exhibiting them to the Academy of Natural Sciences, the members were fully convinced that the facts are such as I have just stated.

It will be observed that these observations were made upon fœtuses of a still earlier period of development, than those quoted from the Cyclopædia of Anatomy and Physiology.

Cellular, or (as it is now called) areolar tissue, is next described, in its mode of production and localities. This is followed with a description of its principal constituents, white and yellow fibrous figures, both of which, according to the American editor, are developed from cells.

Cartilage, fibro-cartilage, synovial membranes, and the connection of the pieces of the skeleton one with another follow in the most natural order. Next comes aponeurology, and then an able section on myology, in which the general anatomy of muscular tissue is admirably displayed. In describing the ultimate fibrilla, Dr. Sharpey observes, that when a single fibril is completely insulated, and highly magnified, it is seen to consist of a row of minute particles connected together like a string of beads. These particles (named "sarcous elements" by Bowman) when viewed with a magnifying power of four hundred or six hundred diameters, appears like little dark quadrangular, and generally rectangular bodies, with bright intervals between them, as if they were connected together by some pellucid substance; but on closer inspection, provided the defining power of the instrument is good, a very faint dark line or shadow will be discovered passing across the fibril in the middle of each of the bright spaces, and sometimes, also, a bright border may be observed on either side of the fibril, so that each of the rectangular dark bodies appears there to be surrounded with a bright area having a similar quadrangular outline, and it may therefore be inferred that the pellucid substance encloses it on all sides. In short, it would appear that the elementary particles of which the fibril is made up, are little masses of pellucid substance, presenting a rectangular outline, and appearing dark in the centre. Their appearance, indeed, suggests the notion of vesicular bodies or cells, cohering in a linear series, the faint transverse marks between being the lines of junction.

With a still higher power the dark central part appears constricted in the middle, or looks as if it consisted of two portions joined together. By altering the focus, the internal dark part becomes light; it is, therefore, evidently transparent, and its dark aspect is probably owing to its refracting the light differently from the surrounding substance.

From this description, it will appear that the "Sarcous Elements" of Mr. Bowman, are in reality cells; the dark spots being the *cavity*, and the pellucid margin the *cell-wall*.

In the arrangement of the sheaths of the muscular fasciculi, the American editor has observed that the filaments of areolar tissue which enter into their composition, for the most part pass in a diagonally crossing manner around the fasciculi, occasionally passing in between the fibres and intermingling with some fine filaments of elastic tissue which are found in this situation. At the extremities of the muscular fasciculi, the filaments of the areolar sheaths become more or less straight, and combine with the fibrous filaments originating in this position to form the tendinous connexion of the muscle. This spiral arrangement of the fibres of the sheath allows the alternate movements of lengthening and shortening in the muscle itself, just as a spiral spring can be alternately increased and diminished in length. Whilst the parallel direction of the fibres of the tendon prevents their elongation, and the necessarily consequent expenditure of the power of the muscle.

The bloodvessels and nerves, involuntary muscles, and chemical properties of muscles are also described in this section, which ends with the consideration of their *vital* properties, under which head will be found a confirmation of Matteucci's Experiments on Electrical Currents in muscles. Indeed, the whole subject of *muscular tissue* may be warmly commended to the careful perusal of the student, as containing one of the best accounts we know of in this interesting department of Physiological Anatomy.

Did time and space allow, we would gladly extend our notice to the other departments of General Anatomy, but enough has perhaps been said to give some idea of the admirable arrangement and execution of the work before us. In the departments of Neurology, the student of Physiological Anatomy will find much to repay a close and careful perusal. The best information on this subject, aided by the observations of the authors themselves, are laid before him, and he will find many of the difficulties, that have heretofore attached themselves to this subject, removed.

The whole work is, in our judgment, the best that has ever issued from the English press, and most admirably adapted, both as a work of reference for the practitioner, and as a text book for the student.

It has been enriched in the department of General Anatomy by the labors of the American editor, who has added many illustrated observations, in some cases original, in others comfirmatory of those already advanced. His reputation as an able microscopist, must greatly enhance the value of the work.

We have said nothing of the Descriptive Anatomy, for reasons already mentioned. Suffice it to say, that praise is not withheld because not merited. It is clear, concise, and yet contains all that is needed to satisfy the tastes of the most fastidious.

---

*An Oration delivered before the South Carolina Medical Association, at its first anniversary meeting, held in Charleston, S. C., February 22d,* 1849. By P. C. Gaillard, M. D.

This well written essay is a pathological argument in favor of Hygienic measures for the prevention of contagious diseases.

The author defines a contagious disease, to be one capable of self-reproduction; and regards as such, all diseases which are communicated by the sick to the well, whether through direct contact, or through the medium of the atmosphere. This definition we conceive to be correctly limited; but there are persons who can perceive no contagion where there is no contact, and who seem to think that fomites have the power of propagating infection as well as contagion, of carrying to distant places portions of atmosphere, pestilential *per se*, and independent of the existence at the place of departure, of the disease so propagated. If there are no facts to serve as a basis for this doctrine, and we doubt that there are any, and if a disease can only be conveyed to another place from that in which it already exists, nothing can be clearer in our apprehension, or a more necessary conclusion of reason, than that extension by such a mode implies the power of self-reproduction in a disease,—implies, in other words, its contagiousness.

Dr. Gaillard insists upon a point too often lost sight of, that *all* of the antecedents to a given event may be necessary to its production, and not a single prominent one of them only. Thus in the case of contagion ; for the disease to be propagated in this manner it is doubtless necessary that the morbid cause should itself possess a certain degree of intensity, that certain atmospheric and other external conditions should be united, and that a certain predisposition should exist on the part of those attacked. Not only must the seed be vigorous, and be received into a congenial soil, but outward influences must favour its germination and subsequent development.

The author takes for granted that there is a *materies morbi* in all contagions, and that this substance being received into the blood, is only, or chiefly injurious there, in so far as it fails to be eliminated by the skin, the intestines, the liver or the kidneys. To illustrate this proposition, he presents a summary of the facts in physiology, which indicate the systems referred to as the outlets of the excrementitious matters produced in the organism, as well as of many hurtful ingesta. This, of course, leads to the conclusion that the material principle of contagious diseases becomes injurious to the economy, only because it was not eliminated with sufficient rapidity; and to this, also, that the best preventive against contagion is a healthy and vigorous state of the excretory apparatus.

But excretion is imperfectly performed under various unfavorable circumstances. If the food is scanty or unwholesome, the supporter of all the functions, the blood, is impoverished ; if clothing is deficient, atmospheric vicissitudes check the depurative action of the skin; if the air is humid, not only is the function of the skin impaired, but the animal temperature is injuriously lowered, and the atmospheric poison is presented to the lungs in a condensed form ; if ventilation is imperfect, not only is the malaria concentrated, but the blood suffers from the want of a due supply of oxygen, and is more directly vitiated by animal and other exhalations; if drainage is defective, deleterious emanations vitiate the sources of life, and moisture works its mischeivous effects; if personal cleanliness is neglected, the wholesome actions of the skin are impeded.

Such is the chain of Dr. Gaillard's argument, in which only one link is defective, that of the substantial nature of the contagious element. In the present state of our knowledge, this proposition has not advanced beyond the stage of hypothesis, one strongly supported, indeed, by analogy and other probable evidence, but still an hypothesis. Upon sound principles of reasoning, therefore, and with a distinct acknowledgement of its character, we are entitled to accept it until a more tangible explanation of the laws of contagion shall be proposed.

It cannot now be denied that a disease will manifest its contagious character just in proportion to the intensity of the unfavorable influences which have been enumerated; and that they or some among them, are just as necessary to the development of its seminal principles, as that light and moisture are to the germination of the seeds of vegetables. Hence, the failure of a disease to be propagated, either by direct or mediate contact, on ordinary occasions, is no argument against its contagious nature, so long as a single example can be produced in which such a mode of propagation is placed beyond reasonable doubt. If one or more persons, sick with such a disease, be conveyed to a distant locality, the malady will extend or not, according to the fitness of the *soil* for its reception. In this way, alone, is it possible to harmonize the discordant opinions entertained by physicians in regard to the contagiousness of cholera and yellow fever.

Dr. Gaillard entertains no doubt, and he supports the opinion by well known facts, that both of these maladies are at times communicable from the sick to the healthy; and he insists upon the necessity which thence arises, of resorting to all those hygienic means which are adapted to prevent the introduction, and limit the ravages of these and kindred diseases. With these conclusions, which appear to rest upon a substantial basis of fact and induction, we most heartily concur.

*Chemical Technology, or Chemistry applied to the Arts and Manufactures.* By Dr. F. Knapp, Professor at the University of Giessen. Translated and edited with numerous notes and additions by Dr. Edmund Ronalds, Lecturer on Chemistry at the Middlesex Hospital, and Dr. Thomas Richardson, of Newcastle-on-Tyne. First American edition, with notes and additions by Professor Walter R. Johnson, of Washington, D. C. Vol. I. Illustrated with two hundred and forty-six engravings on wood. Philadelphia. Lea & Blanchard, 1849.

Though not strictly within our province as medical journalists, we cannot refrain from speaking in terms of commendation of the admirable work with the above title. It forms the second volume on the same subject, and contains descriptions of the various processes employed in the manufacture of Glass, Clay ware and Lime. To those engaged in these manufactures it offers a collection of valuable material not to be met with, we believe, in any work in the English language, and even to those not thus engaged, it presents in an intelligible form information upon subjects of which no one likes to confess ignorance. It is beautifully illustrated and well gotten up, and has been much enriched by the additions of the American editor, who is well known to all engaged in scientific pursuits.

---

*A Theoretical and Practical Treatise on Human Parturition.* By H. Miller, M. D., Professor of Obstetrics and the Diseases of Women and Children in the Medical Department of the University of Louisville. Louisville, John V. Cowling, and George C. Davis: Cincinnati, H. W. Derby & Co.: New York, A. S. Barnes & Co. 1849.

In noticing the above work, our limits will not permit us to enter into an extended analysis of its contents. In the preface, the author gives us some of his reasons for writing a work on obstetrics. His motive in becoming an author, is beyond doubt highly praiseworthy, and we think he has succeeded in laying before the public a good practical work upon obstetrics, written in a style agreeable, concise and perspicuous. In regard to the presentations and positions of the fœtus, the author has adopted the classification of Dugès, "but the nomenclature is his own."

Upon referring, however, to the chapter upon this subject, we really find that his nomenclature is nothing more than the one adopted by other writers on obstetrics. As far as the vertex positions are concerned, the nomenclature of Prof. Miller is identical with that of Moreau. In the other positions there is some little alteration; thus, instead of denominating face positions as right and left *mento*-iliac, they are termed right and left *fronto*-iliac, a change by no means important, and entirely unnecessary. Some similar changes will be found in the designation of pelvic presentations. This is not a matter of much importance, but when an author claims originality for any improvement, he should be careful to ascertain that his claims are well founded.

The description of the mechanism of the various positions is very clear and concise, though its comprehension would be rendered much easier to the student, if the work were illustrated with well executed wood cuts. In a subsequent edition of this valuable treatise, we would suggest the importance of introducing into the body of the work suitable illustrations, at least, of the mechanism of labor.

## Figures and Tables

**Fig. 64. Fig. 65. f1:**